# Mealtime interactions between nursing home staff and residents with dementia: a behavioral analysis of Language characteristics

**DOI:** 10.1186/s12877-023-04320-3

**Published:** 2023-09-23

**Authors:** Wen Liu, Ying-Ling Jao, Anju Paudel, Si On Yoon

**Affiliations:** 1 College of Nursing, The University of Iowa, 50 Newton Rd, Iowa City, IA 52242 USA; 2 Ross and Carol Nese College of Nursing, The Pennsylvania State University, 307B Nursing Sciences Building, University Park, PA 16802 USA; 3 Department of Communicative Sciences and Disorders, New York University, 665 Broadway, New York, NY 10012 USA

**Keywords:** Language, Communication, Mealtime, Nursing home, Dementia, Dyadic interaction, Dyadic research, Secondary analysis

## Abstract

**Background:**

Quality staff-resident communication is crucial to promote outcomes in nursing home residents with dementia requiring assistance during mealtimes. Better understanding of staff-resident language characteristics in mealtime interactions help promote effective communication, yet evidence is limited. This study aimed to examine factors associated with language characteristics in staff-resident mealtime interactions.

**Methods:**

This was a secondary analysis of 160 mealtime videos involving 36 nursing staff and 27 residents with moderately severe to severe dementia in 9 nursing homes. Mixed-effects models was used to examine the relationships between factors and language characteristics in staff-resident mealtime interactions. The independent variables were speaker (resident vs. staff), utterance quality (negative vs. positive), intervention (pre- vs. post-communication intervention), and resident dementia stage and comorbidities. The dependent variables were expression length (number of words in each utterance) and addressing partner by name (whether staff or resident named their partner in each utterance). All models included staff, resident, and staff-resident dyad as random effects.

**Results:**

Staff (utterance n = 2990, 99.1% positive, mean = 4.3 words per utterance) predominated conversations and had more positive, longer utterances than residents (utterance n = 890, 86.7% positive, mean = 2.6 words per utterance). As residents progressed from moderately severe to severe dementia, both residents and staff produced shorter utterances (z=-2.66, p = .009). Staff (18%) named residents more often than residents (2.0%; z = 8.14, p < .0001) and when assisting residents with more severe dementia (z = 2.65, p = .008).

**Conclusions:**

Staff-resident communication was primarily positive, staff-initiated, and resident-oriented. Utterance quality and dementia stage were associated with staff-resident language characteristics. Staff play a critical role in mealtime care communication and should continue to initiate resident-oriented interactions using simple, short expressions to accommodate resident declining language abilities, particularly those with severe dementia. Staff should practice addressing residents by their names more frequently to promote individualized, targeted, person-centered mealtime care. Future work may further examine staff-resident language characteristics at other levels of language using more diverse samples.

## Background

### Person-centered communication in dementia mealtime care

People with dementia in residential care settings (i.e., residents) often exhibit cognitive, functional, and behavioral challenges during mealtime such as disorientation to meal time/place, swallowing difficulties, and resistance to food/care, and require varied levels of support from staff [1]. Mealtime in residential care settings inherently involves dynamic environments, including the staff-resident dyad, as well as the care contexts with multilevel stimuli including meal-related items (e.g., food, drinks, silverware, utensils), other staff and residents, and the physical and social dining environments, which can be chaotic, distracting, and overstimulating for residents with dementia [1, 2].

While mealtime can be challenging for people with dementia, it is a good opportunity to establish and practice pperson-centered interactions and communication. While interacting and communicating with one another, participants reciprocally make an informational exchange through verbal and nonverbal expressions between staff and residents, which can be an important component of person-centered care especially in residential care settings [3–5]. Person-centered communication includes four key elements: (1) recognition (i.e., acknowledging the person with dementia as an individual, such as calling them by their name and integrating their life story and experience into conversations), (2) negotiation (i.e., communication that consults on needs, desires, and preferences of the person with dementia), (3) validation (i.e., feeling-oriented communication that affirms the person living with dementia), and (4) facilitation (i.e., communication used to initiate and sustain interactions) [6]. Person-centered communication helps to facilitate relationship building and positive engagement as well as assess and respond to residents’ needs and preferences. Person-centered communication is especially critical during mealtime – a basic daily activity that not only ensures fundamental health needs including function, hydration, nutritional intake but also fulfills the social needs by providing interpersonal interactions for residents with dementia [5, 7, 8].

### Factors associated with person-centered communication in dementia mealtime care

Multiple factors at the resident-, staff-, and context-levels are associated with person-centered communication between nursing staff and residents with dementia during mealtime. Resident-level factors include social demographics (e.g., age, education), comorbidities, function (e.g., physical and cognitive ability, vision and hearing, swallowing ability/dysphagia), behaviors (e.g., cooperative, neutral, resistive behaviors), verbal communication skills (e.g., language functioning, number of languages speaking), and values (e.g., identity, privacy, autonomy) [9]. Additionally, the neurodegenerative decline in people with dementia particularly affects their linguistic and communicative abilities, such as word finding, conversation initiation and responses, and language comprehension, and may ultimately result in loss of language in the late stage of dementia [10–12]. Staff-level factors include social demographics (e.g., age, sex, race), professional education and experiences (e.g., hours worked per week, qualifications, knowledge of dementia), individual experiences and perceptions (e.g., mood/feelings, perceived workload), verbal and non-verbal communication skills and approaches (e.g., English language skills, encouragement, physical touch), and values (e.g., respecting privacy, preserving identity) [2, 7, 9]. Contextual factors include location (e.g., long-term care facility and unit, dining area), time and duration of care encounters (e.g., beginning/end and duration of mealtime interactions), and environmental stimuli (e.g., background noise/music, temperature) [9].

Mealtime involves dynamic, interactive, and complex interactions among staff-resident dyads. Among the identified multi-level factors associated with person-centered communication in mealtimes, the majority are related to nursing staff and can be modified, illustrating staff’s critical role as active contributors in influencing the quality of communication and addressing the needs and preferences of residents with dementia [9]. Nursing staff are critically positioned in initiating and maintaining successful communication, especially for residents who require mealtime assistance. Staff-level person-centered communication strategies including appropriate assessments of and adaptations to non-modifiable resident-level factors (e.g., cognitive and linguistic capabilities) as well as management of modifiable staff-level factors (e.g., verbal skills and perceptions of resident behaviors) and context-level factors (e.g., environmental stimuli) with institutional support are fundamental in dementia mealtime care to optimize resident outcomes [9, 13–16]. This multilevel perspective of person-centered communication aligns with the recently proposed Person-Centered Communication Enhancement Model (PC-CEM) [17]. The PC-CEM offers a comprehensive theoretical basis for the design, implementation, and evaluation of person-centered communication interventions in dementia care to optimize person-centered communication outcomes at the resident, care provider and system levels [17]. Briefly, to maximize clinical practicality and effectiveness, person-centered communication interventions should be (1) built upon theoretical underpinnings; (2) include components of caregiver communication knowledge and skills development, value-based learning strategies, and ongoing support and feedback; and (3) target interpersonal encounters where an individual’s perspective and abilities are considered and dyadic communications are individualized and adjusted as needed to empower both residents and care providers [17].

### Language analysis in dementia mealtime communication

Verbal communication plays a key role in interacting with one another and achieving desirable outcomes for staff and residents during mealtimes. On that account, understanding the characteristics of language produced during mealtimes is crucial to optimize mealtime interactions as well as behaviors, function, nutrition, and quality of life for residents with dementia. Analysis of language can be at different levels, including word (expression length, word diversity), syntax/sentence (syntactic complexity of sentences), speech (speech rate or intelligibility), and discourse (coherence and cohesion of conversations) [18]. While prior work on dyadic verbal and nonverbal communication behaviors during mealtimes and other activities primarily focused on the quantity (e.g., frequency of utterances and nonverbal behaviors) and quality (staff person-centered vs. task-centered behaviors; resident positive, neutral, vs. challenging behaviors; dyadic positive, neutral, vs. negative interactions) [1, 2, 19, 20], less work has examined language characteristics itself at different levels during dyadic mealtime interactions. Evidence on language characteristics at word-, sentence-, speech- and discourse-levels as well as factors associated with language characteristics during mealtime interactions is limited. Additionally, while prior work suggests strategies to facilitate communication with people with dementia (e.g., reducing speech complexity, producing short sentences, stating resident name to draw their attention, etc.), it focuses on staff-level language characteristics; there is little evidence that considers both staff-resident (dyadic) language characteristics as well as resident individual characteristics such as physical and cognitive status [21]. It is critical to understand dyadic language characteristics during mealtime interactions and the associated factors at the staff and resident levels. Such information will help guide the development and implementation of person-centered mealtime communication interventions to optimize mealtime care and improve resident outcomes including food intake and quality of life.

### Aims

This study aimed to (1) describe word-level language characteristics (i.e., expression length and addressing the partner by their name) in staff and resident utterances during mealtime interactions, (2) examine the associations between staff-level factors (e.g., speaker, utterance quality, staff reception of dementia communication intervention) and language characteristics, and (3) examine the associations between resident-level factors (e.g., speaker, resident dementia stage and comorbidities) and language characteristics.

## Methods

### Study design

This study was a secondary behavioral analysis of videotaped mealtime observations collected from a randomized clinical trial during 2011–2014. The parent trial evaluated the effect of the Changing Talk (CHAT) intervention on staff elderspeak communication and resident resistiveness to care [22]. Elderspeak is a communication style similar to baby talk that features simplistic vocabulary and grammar, shortened sentences, slowed speech, elevated pitch and volume, and inappropriately intimate terms of endearment. It is commonly used by young persons, when communicating with older adults, especially those living in nursing homes (NHs) [22]. The CHAT intervention is a dementia communication training program designed to alert nursing staff to elderspeak communication and its negative effects and to provide supervised practice to facilitate more effective communication, and more information about the intervention is described else [22].

### Samples and settings

In the parent trial, a convenience sample of 127 nursing staff and 83 residents were recruited from 13 NHs in Kansas, USA. NHs located within two hours from the research site and providing care for residents with dementia were recruited. In each NH, residents were eligible if they had (1) a dementia diagnosis of any type or stage based on medical records, (2) staff-reported resistiveness to care, (3) long-stay status, (4) hearing capacity based on medical records (i.e., no hearing deficit with or without assistive hearing devices), and (5) a surrogate decision maker to provide informed consent were eligible [22]. Staff were eligible if they (1) were older than 18 years old, (2) were permanent employees, (3) were able to communicate in English based on staff report, and (4) delivered direct care for a resident participant for at least two times per week over the previous month.

Videos were recorded to capture staff-resident communication during daily care activities, including mealtimes, and were archived in the parent trial. Video recordings were collected on two days at each time point in the parent study. Prior to the first recording session, a practice recording session was completed by a trained research assistant (videographer) using a mini handheld digital video recorder to minimize the Hawthorne effect by (1) allowing residents to adjust to the novelty of being recorded, (2) allowing the videographer to become familiar with daily care routines, (3) evaluating any adverse impact on residents, and (4) establishing behaviors indicating that recording should be discontinued. Recording did not take place during the practice recording session, but the videographer observed and made notes of resident reactions to the presence of the camera and the videographer. After the practice recording session, it was expected that residents would be familiar with videographers’ faces and existence and behave more naturally when formal recordings occurred. Practice recording was not repeated at the remaining data collection points.

Video recordings were collected on day and evening shifts of weekdays. Recordings primarily captured morning care when staff-resident care interactions are most intensive, except when resident resistiveness to care typically occurred at another time of the day based on staff report. The time of day and specific activity recorded were kept constant as much as possible for each resident. Recordings occurred in resident rooms as well as public areas. To facilitate resident de-sensitization to the recording situation, the camera was placed prior to the start of the care activity. Before each recording session, the videographer reminded the resident and staff that recording would take place and obtained oral consent/assent of video recording from the resident and staff. Those recordings that incidentally included persons who did not consent to be in the study were deleted. The videographer stood in an unobtrusive location while the recording occurred, and reviewed video footage on site to ensure the quality of recording. When a resident was recorded during a care activity, including mealtime, the recording started when communication between staff and resident started or continued, and ended when the dyadic communication discontinued or stopped. Therefore, segments of mealtime rather than full mealtime were recorded in the parent study [22].

The archived videos from the parent study were screened for this study. Videos were eligible if they: (1) lasted for ≥ 1 min, (2) captured mealtime interactions between one resident and one staff, and (3) captured utterances with adequate audio quality. A total of 1,748 videos were screened, from which, 1,588 videos were excluded due to lasting < 1 min (n = 63), capturing other activities rather than mealtimes (n = 1,486), involving more than one staff and/or more than one resident (n = 34), and poor audio quality (n = 5). Thus, 160 videos were eligible for this study, of which, 110 were collected prior to the staff communication training (pre-intervention) and 50 were after the training (post-intervention) [22].

### Data coding

In this study, staff and resident utterances were transcribed and coded using the refined Cue Utilization and Engagement in Dementia (CUED) Mealtime Video-Coding Scheme in 2019 using Noldus Observer® 14.0 (Noldus Information Technology Inc., Leesburg, VA, USA) [23]. In the refined CUED, there were eight codes representing positive utterances (i.e., asking for help/cooperation, assessing for comfort/condition, giving choices, orientation/giving instructions, showing approval/agreement, showing interest, gain attention verbally, others) and four codes representing negative utterances (i.e., interrupting/changing topic, verbal refusal/disagreement, controlling voice, others). The refined CUED showed evidence of feasibility, ease of use, and adequate inter-coder reliability among the four trained coders using 22 videos randomly selected from the sample (Cohen’s Kappa = 0.93–0.97, 95% CI = 0.91–0.98, ±1s tolerance), and adequate predictive and construct validity [2, 23, 24].

Four coders were trained by the first author following a standard training and coding manual. After establishing inter-coder reliability, each of the four trained coders coded a subset of videos independently. Each utterance (a statement or question) was assigned a code. All utterances were coded as point events, where onset time (vs. offset time) of utterance was coded. Detailed coding process and conceptual and operational definitions of all codes are described elsewhere [2, 23, 24]. Coded data were exported from the Noldus Observer® to Excel worksheets.

#### Dependent variables

For this study, two dependent variables—expression length and addressing partner by their name— representing word-level language characteristics were coded for each transcribed utterance of residents and staff.

- *Expression length*, a continuous variable operationalized as the number of words produced in each utterance.
- *Addressing the partner by their name*, a binary variable operationalized as whether staff or resident named their dyadic partner (i.e., resident or staff) in each utterance.

#### Independent variables

For this study, the independent variables included speaker, utterance quality, and intervention, in addition to resident dementia stage and comorbidities.

- *Speaker*, a binary variable operationalized as whether resident or staff produced each utterance.
- *Utterance quality*, a binary variable operationalized as whether each utterance was coded as positive vs. negative in quality based on the refined CUED.
- *Intervention, a* binary variable operationalized as whether videos were collected before or after the dementia communication training was delivered to staff (pre- vs. post-CHAT intervention).

### Data analysis

Characteristics of facilities, staff, residents, and utterances were described using descriptive statistics (n/%, mean/SD). Poisson-link mixed-effects model was used to fit expression length with speaker (resident vs. staff), utterance quality (negative vs. positive), and intervention (pre- vs. post-intervention) as fixed effects. Poisson distribution is appropriate as the dependent variable, expression length, is a count of the number of occurrences during a defined time interval [25]. Logit-link mixed-effects model was used to fit the binary measure of whether the speaker addressed the partner by their name with speaker, utterance quality, and intervention as fixed effects. Further, resident dementia stage and comorbidities (log-transformed) were added to the models to examine the associations between resident characteristics and language and whether estimates of other fixed effects change. All models included staff, resident, and staff-resident dyad as random effects and were fit using the LMER package in R version 4.1.0 [26]. The level of significance was set as 0.05.

### Ethical considerations

#### Ethical approval

s were obtained through Institutional Review Boards of universities where the studies were conducted. In the parent study, NHs were first enrolled and randomized to the intervention or waiting list control group. In each enrolled NH, staff and resident participants were provided with information about the study and recruited with written consent (staff) and surrogate consent from the resident’s Legally Authorized Representative and resident assent (resident) [22].

This study as a secondary data analysis of the videos collected from the parent study was approved by Institutional Review Board (IRB#: 201,208,797, see Supplementary file #1). A data use agreement between the institution of parent study PI and the institution of the secondary data analysis study PI (first author of this manuscript) was established (see Supplementary file #2), indicating the approved use of videos from residents and staff who indicated consent in the use of their video recordings in future research studies in their written consent forms. All videos used in the study were stored in the Research Data Storage Service (RDSS) at the University of Iowa, a secured system for backups, archiving, and storing research data files in the institution system.

## Results

### Video sample characteristics

The 160 videos lasted between 1 and 23.8 min (mean = 4.5, SD = 3.8). Most of the videos were recorded in the dining rooms (N = 147, 92%) and the rest were recorded in the residents’ own room (N = 13, 8%). In three quarters of the videos (N = 120, 75%), residents were seated in a chair, and in the other 25% of the videos, residents were seated in a wheelchair. The 160 videos that were used in this study involved 27 residents and 36 nursing staff (53 unique staff-resident dyads) in 9 NHs. In this study, while each resident was assisted by one staff in each videotaped mealtime interaction, the resident could be assisted by the same or different staff across all the videorecorded mealtime interactions, resulting in one or multiple unique staff-resident dyads. Among the 27 residents, 9 residents, respectively, were assisted by 1 staff in the videos that captured the residents, resulting in 9 unique staff-resident dyads; 12 residents, respectively, were assisted by 2 different staff in the videos that captured the residents, resulting in 24 unique staff-resident dyads; 4 residents, respectively, were assisted by 3 different staff in the videos that captured the residents, resulting in 12 unique staff-resident dyads; 2 residents, respectively, were assisted by 4 different staff in the videos that captured the residents, resulting in 8 unique staff-resident dyads.

### Facility and participant characteristics

The nine NHs ranged from 43 to 163 beds in size (Median = 60 beds) and were distributed evenly on location (n = 4 rural, n = 5 urban), profit status (n = 4 for-profit, n = 5 non-for-profit), and quality ratings (n = 4 rated1-3 stars, n = 5 rated 4–5 stars). Five NHs had one or more memory care units.

Resident characteristics were collected through medical records. As show in Table 1, resident participants had a mean age of 85.6 years old. All residents were White. The majority were female (63.0% vs. 37% male) and non-Hispanic (92.6% vs. 7.4% Hispanic). Residents had moderately severe (70.0%) or severe (30.0%) dementia as determined by reviewing Minimum Data Set (MDS) 3.0 using the Functional Assessment Staging in Alzheimer’s Disease (FAST, total score ranges from 1, normal cognition, to 7, severe dementia) [27]. Residents had moderate levels of physical comorbidities (mean = 27.1, range = 19–36) as evaluated by reviewing MDS 3.0 and clinical records using the Modified Cumulative Illness Rating Scale (total score ranges from 0 to 70 with higher scores indicating more comorbidities) [28].

**Table 1:** Resident characteristics

Continuous variables	N*	M ± SD	Range
Resident age (years)	26	85.6 ± 8.6	64.0–104.0
Resident physical comorbidities (0–70)	24	27.1 ± 5.3	19.0–36.0
Categorical variables	N	n	%
Resident gender	27		
Male		10	37.0
Female		17	63.0
Resident race: White	27	27	100.0
Resident ethnicity	27		
Non-Hispanic		25	92.6
Hispanic		2	7.4
Resident dementia stage	20		
Moderately severe dementia (FAST = 6)		14	70.0
Severe dementia (FAST = 7)		6	30.0

* The N of some resident characteristics is less than 27 due to missing

Staff characteristics were collected using self-report surveys. As shown in Table 2, staff participants had a mean age of 35.9 years old, worked as a caregiver for a mean length of 9.5 years, and worked at the current NH for a mean length of 4.0 years (Table 2). Most staff were female (80.6% vs. 19.4% male), non-Hispanic (75.0% vs. 25% Hispanic), and White (75.0% vs. 25% African American), had completed or were attending college (72.2% vs. 27.8% completed high school). The majority were certified nursing assistants (85.7%) and the rest were registered nurses (5.7%) and licensed practical nurses (8.6%).

**Table 2:** Staff characteristics

Continuous variables	N	M ± SD	Range
Staff age (years)	36	35.9 ± 12.4	19.0–79.0
Staff years working as caregiver	36	9.5 ± 8.6	0.3–31.0
Staff years working in current facility	36	4.0 ± 3.7	0.1–13.0
Categorical variables	N	n	Percentage (%)
Staff gender	36		
Male		7	19.4
Female		29	80.6
Staff race	36		
White		27	75.0
African American		9	25.0
Staff ethnicity	36		
Non-Hispanic		27	75.0
Hispanic		9	25.0
Staff education	36		
High school		10	27.8
College		26	72.2
Staff job title	35*		
Certified nursing assistants		30	85.7
Registered nurse		2	5.7
Licensed practical nurse		3	8.6

* The N of staff job title is less than 36 due to missing

### Language characteristics

Staff (utterances n = 2990) spoke three times more often than residents (utterances n = 890, Table 3). Most utterances produced by staff (99.1%) and residents (86.7%) were positive. Staff produced longer expressions (mean = 4.30 words per utterance) than residents (mean = 2.64 words per utterance) in general, as well as in both positive and negative utterances (4.31 and 3.58 words in staff utterances vs. 2.53 and 3.37 words in resident utterances). Staff addressed residents by their name (18%) more often than residents naming staff (2%) in general, as well as in both positive and negative utterances (18% and 14.8% of staff utterances vs. 1.8% and 3.4% of resident utterances).

**Table 3:** Resident and staff language characteristics by speaker, pre- and post-intervention, and utterance quality

Language characteristics	Resident			Staff		
	Overall	Pre-intervention	Post-intervention	Overall	Pre-intervention	Post-intervention
Categorical variables	N (%)			N (%)		
Total number of utterances	890	659	231	2990	2143	847
Utterance quality						
Negative	118 (13.3)	98 (14.9)	20 (8.7)	27 (0.9)	16 (0.7)	11 (1.3)
Positive	772 (86.7)	561 (85.1)	211 (91.3)	2963 (99.1)	2127 (99.3)	836 (98.7)
Total number of utterances including partner name	18 (2.0)	10 (15.2)	8 (3.5)	537 (18.0)	368 (17.2)	169 (20.0)
Negative	4 (3.4)	3 (3.1)	1 (5.0)	4 (14.8)	2 (12.5)	2 (18.2)
Positive	14 (1.8)	7 (1.3)	7 (3.3)	533 (18.0)	366 (17.2)	167 (20.0)
**Continuous variables**	**Mean (SD)**			**Mean (SD)**		
Length of expression	2.64 (2.27)	2.79 (2.38)	2.24 (1.88)	4.30 (2.98)	4.25 (3.01)	4.42 (2.90)
Negative	3.37 (3.00)	3.70 (3.14)	1.89 (1.56)	3.58 (2.28)	2.87 (1.51)	4.55 (2.84)
Positive	2.53 (2.12)	2.64 (2.19)	2.27 (1.90)	4.31 (2.98)	4.26 (3.01)	4.42 (2.90)

### Expression length

**Fig. 1:**
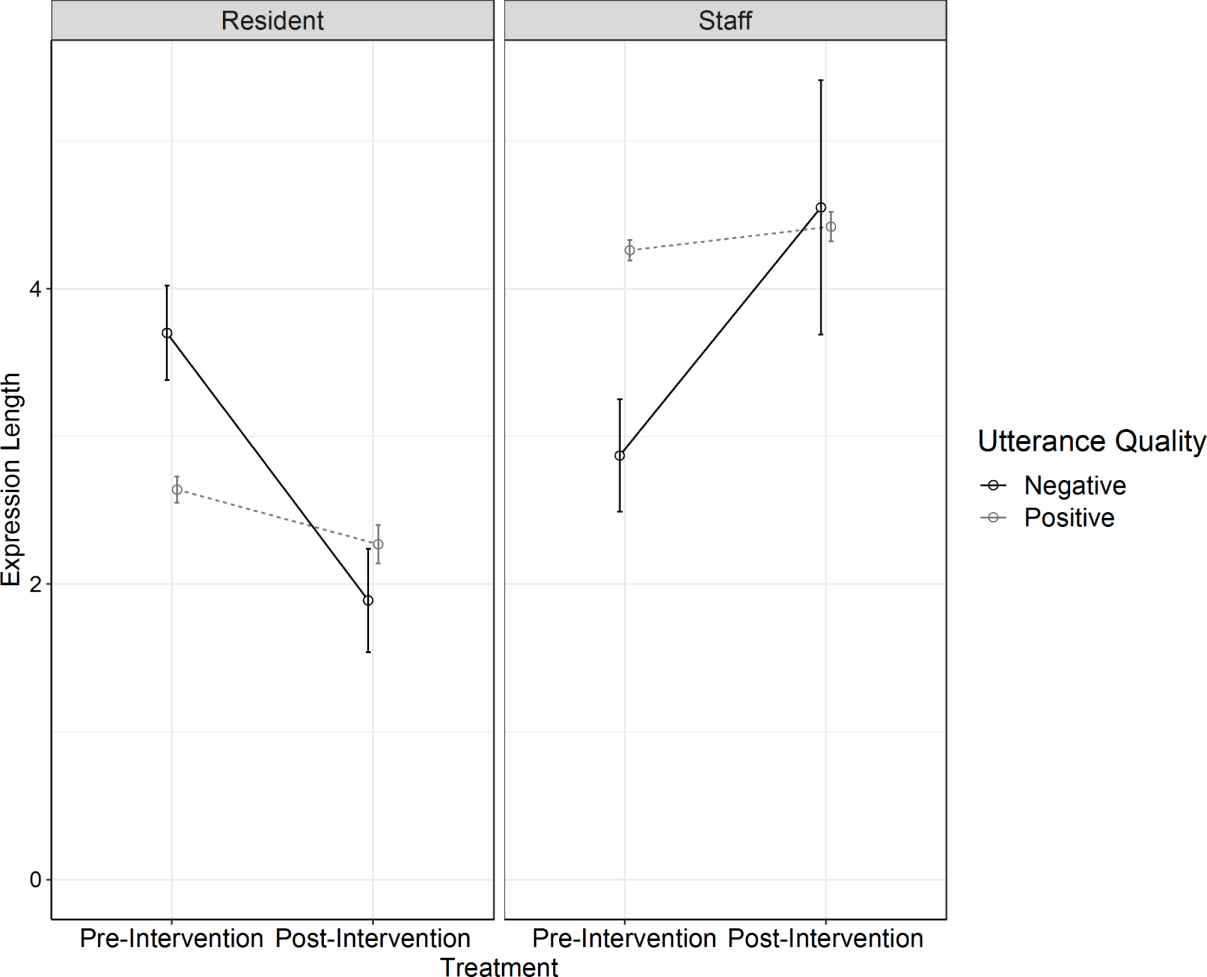
Expression length as a function of speaker, intervention and utterance quality. Note: Error bars indicate the standard errors of the mean

The model revealed expression length was significantly associated with speaker (z = 21.67, p < .0001) and utterance quality (z = 2.00, p = .046; Table 4), indicating that staff generally produced longer expressions than residents, and positive utterances were longer than negative utterances. Expression length was not significantly associated with intervention (z=-1.10, p = .27). The three-way interaction between speaker, utterance quality, and intervention was significant (z = 4.74, p < .0001), which was driven by the significant interaction between speaker and utterance quality pre-intervention only (Fig. 1). Particularly, staff positive utterances were longer than their negative utterances (z = 2.80, p = .005), whereas resident positive utterances were shorter than their negative utterances pre-intervention (z=-6.20, p < .0001). The interaction between speaker and utterance quality was not significant post-intervention (z=-1.36, p = .17).

After adding resident comorbidities and dementia stage, the association with speaker (z = 18.68, p < .0001) and utterance quality (z = 2.20, p = .03), and the three-way interaction between speaker, utterance quality, and intervention (z=-3.82, p < .001) remained significant. Expression length was significantly associated with resident dementia stage (z=-2.66, p = .008), suggesting that as resident dementia stage progresses from moderately severe into severe, both residents and staff are likely to produce shorter utterances. Expression length was not significantly associated with resident comorbidities (z = 1.75, p = .08).

**Table 4:** Role of speaker, utterance quality, and intervention, and resident characteristics on expression length

Fixed effects	Model w/o resident characteristics				Model with resident characteristics			
	Estimate	SE	*z*	*p*	Estimate	SE	*z*	*p*
(intercept)	1.32	0.04	31.99	< .0001	5.47	1.91	2.87	0.004
**Speaker (resident vs. staff)**	**0.56**	**0.03**	**21.67**	**< .0001**	**0.51**	**0.03**	**18.68**	**< .0001**
**Utterance Quality (negative vs. positive)**	**0.19**	**0.09**	**2.00**	**0.046**	**0.24**	**0.11**	**2.20**	**0.03**
Intervention (pre- vs. post-intervention)	-0.05	0.04	-1.10	0.27	-0.07	0.04	-1.68	0.09
Speaker * Utterance Quality	**0.49**	**0.14**	**3.61**	**< .001**	**0.56**	**0.16**	**3.54**	**< .001**
Speaker * Intervention	**0.13**	**0.06**	**2.18**	**0.03**	**0.19**	**0.06**	**3.19**	**0.001**
Utterance Quality * Intervention	-0.23	0.17	-1.36	0.17	-0.32	0.19	-1.69	0.09
Speaker * Utterance Quality * Intervention	**-1.06**	**0.28**	**-3.75**	**< .001**	**-1.16**	**0.31**	**-3.82**	**< .001**
Resident comorbidities					0.33	0.19	1.75	0.08
**Resident dementia stage**					**-2.69**	**1.01**	**-2.66**	**0.008**
Random effects	Variance	SD			Variance	SD		
Staff (Intercept)	0.02	0.14			0.02	0.14		
Resident (Intercept)	0.02	0.13			0.01	0.07		
Dyad (Intercept)	0.01	0.12			0.02	0.12		

Note. N = 158 videos. The dependent measure is the number of words produced in each utterance (expression length, continuous variable). Speaker [Resident (-0.77) vs. Staff (0.23)], Utterance Quality [Negative (-0.96) vs. Positive (0.04)], and Intervention [pre- (-0.28) vs. post-intervention (0.72)] are coded as mean-centered fixed effects. SE = Standard Error. Values in bold indicate significant results (p < .05)

### Addressing the partner by their name

The model revealed addressing the partner by their name was significantly associated with speaker (z = 8.14, p < .0001), indicating staff addressed residents by their name more often than residents addressing staff by their name during mealtimes (Table 5). Utterance quality (z = 0.21, p = .84) and intervention (z = 1.79, p = .07) were not significantly associated with the frequency of addressing partner by name. After adding resident comorbidities and dementia stage, speaker (z = 4.86, p < .0001) and dementia stage (z = 2.65, p = .01) were significantly associated with addressing partner by name. In planned comparisons, staff and resident utterances were analyzed separately. The association between dementia stage and staff addressing the resident by their name remains significant (z = 3.05, p = .002), whereas the association between dementia stage and resident addressing the staff by their name was not significant (z=-0.68, p = .50). Staff addressed their partner (i.e., resident) by their name more frequently than residents as resident dementia stage progresses from moderately severe into severe. The association between resident comorbidities and addressing the partner by their name was not significant (z=-0.98, p = .33).

**Table 5:** Role of Speaker, Utterance Quality, Intervention, and Resident Characteristics on Addressing Partner by Name

Fixed effects	Model w/o resident characteristics				Model with resident characteristics			
	Estimate	SE	*z*	*p*	Estimate	SE	*z*	*p*
(intercept)	-2.27	0.18	-12.36	< .0001	-21.66	8.05	-2.69	0.007
**Speaker (resident vs. staff)**	**2.33**	**0.29**	**8.14**	**< .0001**	**3.00**	**0.62**	**4.86**	**< .0001**
**Utterance Quality (negative vs. positive)**	0.10	0.49	0.21	0.84	2.35	3.09	0.76	0.45
Intervention (pre- vs. post-intervention)	0.41	0.23	1.79	0.07	0.52	0.30	1.74	0.08
Speaker * Utterance Quality	1.25	0.85	1.48	0.14	-8.62	13.09	-0.66	0.51
Speaker * Intervention	-0.50	0.54	-0.93	0.35	-1.22	0.94	-1.30	0.19
Utterance Quality * Intervention	-0.25	0.92	-0.27	0.79	-3.42	4.32	-0.79	0.43
Speaker * Utterance Quality * Intervention	-0.40	1.71	-0.23	0.82	13.41	18.09	0.75	0.46
Resident comorbidities					-0.94	0.96	-0.98	0.33
**Resident dementia stage**					**11.60**	**4.37**	**2.65**	**0.01**
Random effects	Variance	SD			Variance	SD		
Staff (Intercept)	0.32	0.57			0.36	0.60		
Resident (Intercept)	0.31	0.56			0.38	0.62		
Dyad (Intercept)	0.19	0.44			0.15	0.38		

Note. N = 158 videos. The dependent measure is whether the utterance includes the partner’s name (addressing partner by name, binary variable). Speaker [Resident (-0.77) vs. Staff (0.23)], Utterance Quality [Negative (-0.96) vs. Positive (0.04)], and Intervention [pre- (-0.28) vs. post-intervention (0.72)] are coded as mean-centered fixed effects. SE = Standard Error. Values in bold indicate significant results (p < .05)

## Discussion

This study described staff-resident language characteristics (i.e., expression length, addressing the partner by their name) during mealtime interactions and examined their associations with speaker, utterance quality, staff reception of dementia communication intervention, and resident characteristics (i.e., dementia stage, comorbidities). While staff predominated dyadic conversations, residents were also involved in the conversations. While both staff and residents predominately used positive communication, staff used more positive communication comparatively. Not surprisingly, staff spoke longer sentences and named their partner (i.e., resident) more often than residents, indicating staff-resident mealtime interactions were primarily staff-initiated and resident-oriented. Most of the mealtime interactions in this study occurred in the dining areas (vs. resident own room), representing common mealtime care practices in NHs. Residents were exposed to different environmental stimuli including food, conversations, and assistance provided by staff as well as occupied with eating food, and therefore, were less dominant in dyadic interactions.

Interestingly, staff produced longer utterances in their positive (vs. negative) utterances while residents produced longer utterances in their negative (vs. positive) utterances; however, the difference between positive and negative utterances in both staff (0.87 words) and residents (-0.84 words) was small. Such findings may be due to the unbalanced distribution of positive vs. negative utterances in staff and residents. Utterance quality in the study sample had limited variations (99.1% positive and 0.9% negative in staff utterances, 86.7% positive and 13.3% negative in resident utterances). Further analysis indicated that staff positive utterances (n = 2963) were distributed across all eight codes (see Data Coding section) with varied frequencies (ranging from 927 to 52) and were primarily coded as orientation/giving instructions (n = 927, 31.3%) which may require more words, while resident positive utterances (n = 772) were primarily showing interest (n = 294, 38.1%) and approval/agreement (n = 265, 34.3%) which may only require fewer words [29]. Future work needs to examine the role of speaker and utterance quality on expression length using more diverse samples.

The study showed that staff and resident expression length was associated with resident dementia stage. This is consistent with prior work that residents with neurodegenerative deterioration produce more frequent, easier words and shorter, simpler sentences [10, 12]. Residents with dementia often experience communicational and discourse breakdowns due to cognitive and linguistic impairments, and are unable to adjust their expressions depending on the communicative contexts [30]. They are less likely to understand what information and which level of detail is appropriate to convey to their staff partners, and are only able to produce short, simple, and possibly repeated words due to their word retrieval difficulties and conversational inefficiency. Further, residents demonstrate progressive declines at both basic and complex levels of language (e.g., word, phrases, sentences, grammar) as their dementia stage progresses, such as difficulties with naming and verbal fluency, reduced phrase length, impaired phrase repetition, and reduced sentence generation and construction [31]. For example, residents with severe dementia may only be able to speak approximately a half-dozen intelligible different words or fewer, or repeatedly use a single intelligible word over a day, a conversation, or a care interaction [27].

The study showed that staff expressions became shorter and simpler when providing mealtime care to residents at the stage of severe (vs. moderately severe) dementia, possibly because staff adapted their communication to accommodate residents’ decline in understanding and mastery of language due to residents’ cognitive decline. Staff as cognitively intact individuals were able to adjust expressions based on their partners’ needs in social interactions, such as using shorter, simpler phrases in communicating with residents with dementia [30]. The study findings support the Person-Centered Communication Enhancement Model (PC-CEM) that care providers consider resident abilities and needs during interpersonal communication and modify communication as necessary [17]. Our study findings are also consistent with prior reports that conversational supports targeting care activities and the resident partner such as using repeated, continuing verbal cues are useful and effective strategies in managing mealtime challenges [32] and improving eating performance [33, 34] in residents. A recent review also suggested that respect of resident care needs and communication ability and the use of a flexible and adapted communication approach matching resident language ability are important factors associated with communication improvement between nursing staff and people with dementia [9]. Therefore, besides accommodating residents’ declining linguistic abilities, staff should be aware of residents’ remaining capabilities and strengths in communication and provide linguistically stimulating environments that can facilitate implicit and effortless learning among residents during social events (e.g., mealtimes) to assist with their linguistic abilities.

Staff named residents more often as resident progresses from moderately severe to severe dementia stage. Addressing residents by their name during dyadic communication is a critical strategy of person-centered care to acknowledge resident identity, show respect, and establish emotional/personal connection, as well as to engage residents in activities [35]. While addressing residents by their name in dyadic communication has been a highly recommended, simple, resident-centered care strategy [36], our findings indicated less than 20% of staff utterances called their resident partner’s name during mealtimes, indicating urgent needs for improvement of the use of this strategy in practice. One possible explanation why addressing the partner by name was not associated with utterance quality (i.e., quality of verbal communication) might be due to this low number of utterances involving calling a partner by name. Prior work has also reported mixed findings on the associations between addressing resident by their name and communication [37, 38], future research needs to examine their associations in larger, diverse samples.

Staff intervention to avoid elderspeak was not associated with staff-resident language characteristics in this study, possibly because the dementia communication training tested in the parent trial focused on reducing elderspeak (i.e., babytalk to older adults) by staff when communicating with residents during care activities in general, not necessarily focusing on other communication approaches or activities during mealtimes [22]. A similar analysis based on communication studies that specifically focused on improving mealtime interactions might yield different findings and could be considered for future work. In addition, resident comorbidities were not associated with staff-resident language characteristics in this study. However, findings were partially consistent with prior work that reported mixed findings on the associations between communication and resident comorbidities (i.e., negative and no associations) [39]. Future examination of the role of comorbidities is needed.

The sample in the parent clinical trial focused on residents with staff-reported resistiveness to care during daily activities, which is a population that may require additional attention in dyadic communication. Resident resistive behaviors are considered a way to communicate their needs, preferences, and wants and maybe the only way of communication for residents who cannot verbalize or have lost their language ability. Recent work showed that the use of person-centered verbal cues was associated with increased food intake among residents who were compliant, and were associated with decreased intake among those with resistive behaviors during mealtimes [8]. Residents showing resistiveness usually indicate dissatisfaction with the provided care or food, and may require additional support from staff beyond simple cues and calling their names [8]. While confronting restiveness to care from residents, staff reported experiences of discomfort as well as reflections on their own attitudes and behaviors as well as approaches that may help them manage and eventually reduce their discomfort [40]. Meanwhile, staff reported the use of strategies, including reconceptualizing and understanding the meaning and underlying reasons for resistiveness to care, stepping back for a while to reduce the tension, accepting resistiveness to care as a way to communicate needs rather than disrupting mealtimes, and providing continuous support or reapproaching the resident at a later time as appropriate [40]. This study did not consider the role of resistiveness to care because all residents had staff-reported resistiveness to care, and future research may consider examining the impact of resistive to care on language characteristics.

While current mealtime care practice in NHs may evolve due to various factors, such as the shift of care from task-oriented to person-oriented, the COVID-19 pandemic and its following consequences, and the understaffing, the video sample collected between 2011 and 2014 reflects current dyadic mealtime care interactions to certain degrees for three reasons. First, the videos reflect the nature of the complex and dynamic staff-resident mealtime verbal interactions that feature different levels of recognition, negotiation, validation, and facilitation. Second, the CUED tool used to assess staff and resident utterance during mealtime interactions was developed based on extensive systematic reviews of literature on dementia mealtime care research including person-centered and task-centered care measures [23, 41, 42]. Lastly, the dyadic mealtime interactions captured in the videos reflect current NH mealtime care practices. For example, staff verbally engaged residents using primarily positive strategies (99.1%) and 86.7% of residents’ utterances were positive in the study. The findings are consistent with a prior report that identified 96% of staff verbal and nonverbal behaviors as positive during mealtime care to NH residents with dementia [20] and another recent study that reported 83.8% of dyadic interactions were positive with the rest being neutral (10.8%) or negative (5.4%) among staff and cognitively impaired residents during various daily care activities [19].

### Strengths

The use of videotaped observations and a validated behavioral tool in this study captured the complexity and interactivity of dyadic mealtime interactions. The use of videos allows for repetitive reviews of dyadic verbal interactions for precise and accurate coding and in-depth analysis of utterance and language characteristics. The CUED is developed based on extensive literature review of dementia mealtime care research and captures a comprehensive list of staff and resident verbal behaviors from both positive and negative perspectives, addressing the current gaps in characterizing dyadic mealtime verbal communication.

### Limitation

The video sample captured primarily segments of meals (vs. full meals) and 1:1 (vs. 1:2, 2:1, or other complex) interactions. Future research may examine staff-resident language characteristics using full-meal observations that capture varied dynamic complexity of mealtime interactions. Further, this study primarily focused on the analysis of word-level language characteristics of staff and residents because utterances of nursing staff and residents with dementia in this study were short and simple, and conversations jumped from one topic to another due to the nature of dynamic and fluid dyadic mealtime interactions, which makes analysis at other language levels (e.g., syntax and discourse level) challenging. Future work may expand the analysis to other levels of language (e.g., syntactic and/or discourse level) using more diverse samples.

While videos were mostly collected during morning care in the parent study, the time of the meal were not recorded, and therefore the role of different types of meals (breakfast, lunch, dinner) could not be examined. Videotaped observations collected from pre- and post-intervention were used in this study. While measures (e.g., practice recording session) have been taken to minimize the Hawthorne effect of videotaping on staff-resident interactions, staff may perform differently from usual care due to social desirability. The video sample may not reflect the most current mealtime care practice and future work can validate the findings using up-to-date mealtime observations.

Lastly, staff participants were primarily nursing care providers and resident participants had exclusively moderately-severe to severe dementia with staff-reported resistiveness to care in NHs. Therefore, findings have limited generalizability to other staff-resident populations in other care settings.

## Conclusion

Quality dyadic communication is crucial to promote care quality as well as resident behaviors, function, hydration, and nutrition during mealtimes. This study provided preliminary evidence on the associations of staff-resident language characteristics with utterance quality and resident dementia stage. Residents with dementia have limited language ability which influences the complexity and length of conversations they can initiate and understand. Our findings indicate there is room to increase the use of person-centered care strategies including simple, short expressions and addressing residents by name during mealtime care practice of people with dementia. Future work needs to examine staff-resident language characteristics at the word and other levels of language using larger, diverse samples such as full-meal observations.

## Data Availability

Video observations are identifiable data and will not be open to public due to privacy/ethical reasons. Non-identifiable data including coded data from videos that support the findings of this study are available from the corresponding author upon reasonable request.
